# ^87^Sr/^86^Sr evidence from the epeiric Martin Ridge Basin for enhanced carbonate weathering during the Hirnantian

**DOI:** 10.1038/s41598-017-11619-w

**Published:** 2017-09-12

**Authors:** Dongping Hu, Xiaolin Zhang, Lian Zhou, Stanley C. Finney, Yongsheng Liu, Danielle Shen, Megan Shen, Wei Huang, Yanan Shen

**Affiliations:** 1 School of Earth and Space Sciences, University of Science and Technology of China, Hefei, 230026 China; 2 State Key Laboratory of Geological Processes and Mineral Resources, Faculty of Earth Sciences, China University of Geosciences, Wuhan, 430074 China; 3 Department of Geological Sciences, California State University at Long Beach, Long Beach, CA 90840 USA; 4 University of Maryland, College Park, MD 20742 USA

## Abstract

A pronounced positive δ^13^C excursion in the Hirnantian Age has been documented globally, reflecting large perturbations of carbon cycling in the Late Ordovician oceans. Increased organic-carbon burial or enhanced carbonate weathering during glacioeustatic sea-level regression has been proposed to account for this anomalous C-isotope excursion. To test the two competing hypotheses, we measured ^87^Sr/^86^Sr and δ^13^C of carbonates from the Copenhagen Canyon section in Nevada, USA. Our data reveal two rapid negative ^87^Sr/^86^Sr shifts that coincide with two prominent positive δ^13^C excursions and glacial advances. Numerical model simulations suggest that enhanced weathering of carbonates driven by glacio-eustatically controlled sea-level fall is required to produce the observed drops of ^87^Sr/^86^Sr and the coeval large positive δ^13^C excursions, possibly with or without increased organic carbon burial.

## Introduction

The Late Ordovician was a time interval of continental glaciation developed in the Southern Hemisphere that coincided with a mass extinction event during the Hirnantian Age^1, 2^. Thus, the Hirnantian extinction event has been attributed to glaciation-induced environmental and climatic deteriorations and changes in ocean chemistry^2–10^. The Hirnantian Stage also records a concurrent positive δ^13^C excursion (up to ~7‰) observed globally both in carbonates and organic carbon^1, 3, 8, 10–15^, reflecting global perturbation of carbon cycle in Late Ordovician oceans. Two competing hypotheses have been proposed to explain the Hirnantian positive C-isotopic excursion termed HICE. It has been hypothesized that HICE may have resulted from a large increase in primary productivity and burial of organic carbon in the deep ocean^3, 4^. The enhanced burial of organic carbon could also have simultaneously promoted a drawdown of atmospheric *p*CO_2_ and ultimately led to the glaciation^3, 4^. However, organic-rich Hirnantian sedimentary rocks have not been identified^12, 13^, and the HICE would have predated the glacio-eustatic sea-level fall, had it been caused by enhanced burial of organic carbon and drawdown of atmospheric *p*CO_2_^11, 13^. In fact, organic-rich sedimentary rocks and thus enhanced burial of organic carbon characterize uppermost Katian strata in deep marine sections in Nevada^5, 11^. Alternatively, Kump *et al*.^16^ hypothesized that the enhanced weathering of carbonate platforms that were exposed during glacioeustatic sea-level lowstand could have produced the HICE. According to the weathering hypothesis, a long-term drawdown of atmospheric *p*CO_2_ through increased weathering of silicate rocks could have led to the Hirnantian glaciation^16^. However, it has been argued that increased weathering of silicate rocks could not have led to the Hirnantian glaciation because the glaciation would first have to cause the sea-level fall that would expose silicate rocks to enhanced weathering^12^.

**Figure 1:**
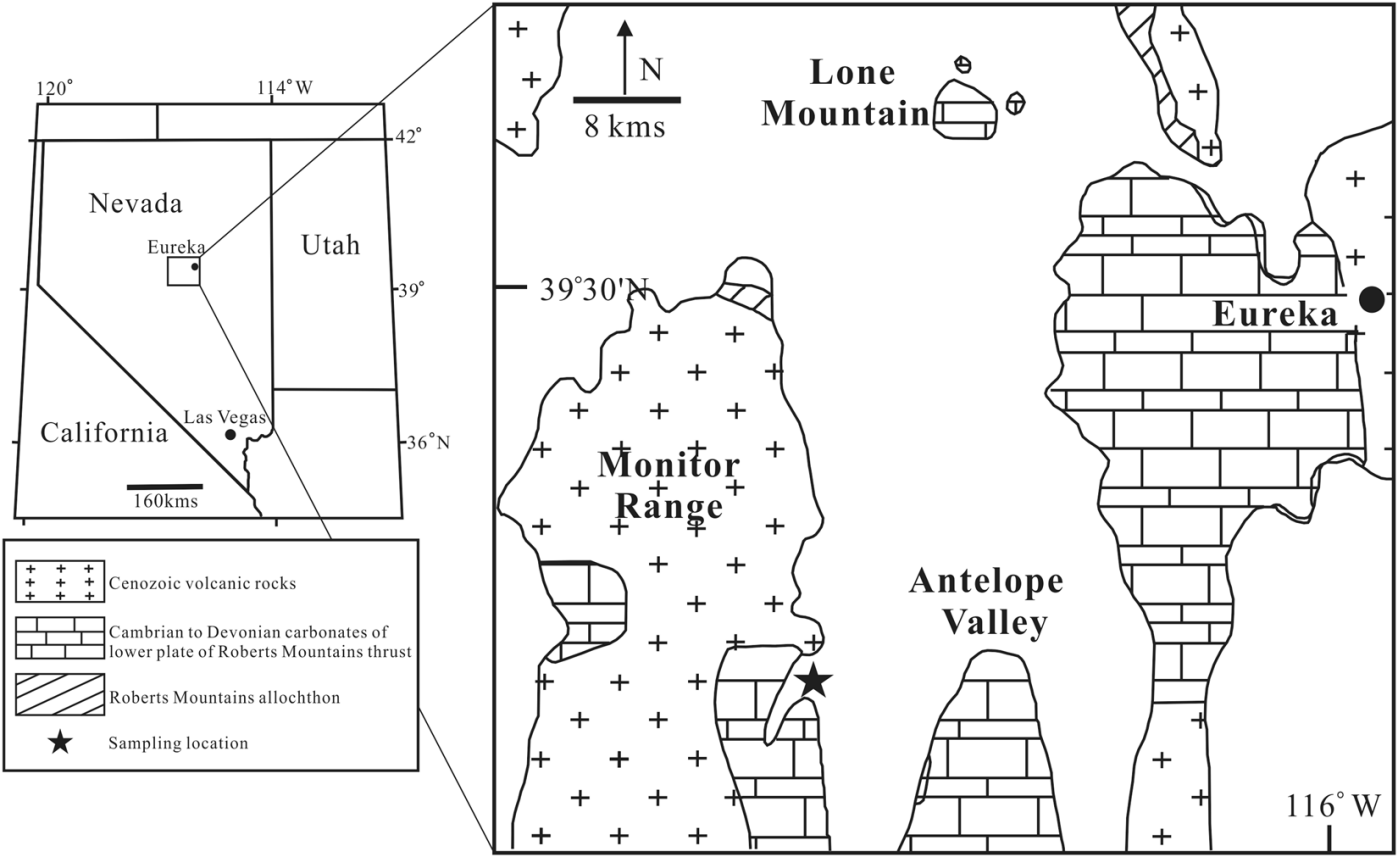
Index map showing location of Copenhagen Canyon section in central Nevada, USA (modified from Finney *et al*.^5^).

The interpretation of the HICE is important in our understanding of the causes of the Late Ordovician glaciation and paleo-environment in which the mass extinction occurred. In this study, we carry out high-resolution analyses of ^87^Sr/^86^Sr and δ^13^C for carbonates from the Copenhagen Canyon section in central Nevada, USA (Fig. 1). ^87^Sr/^86^Sr has been widely used to constrain continental weathering intensity in the geological past^17–21^. In particular, the relationships between marine ^87^Sr/^86^Sr ratios and glaciations have shown how glaciation, as a long considered effective cause of increasing weathering rates, could change the ^87^Sr/^86^Sr ratios of seawater^22–28^. The Ordovician ^87^Sr/^86^Sr of carbonates have provided insights into paleo-climatic changes^29–33^. However, few high-resolution ^87^Sr/^86^Sr measurements on the Hirnantian carbonates have been carried out to reconstruct continental weathering history during the glaciation. In this study, we report δ^13^C and ^87^Sr/^86^Sr data of carbonates from the Copenhagen Canyon section. Integrated with numerical modeling, our results can be used to test the competing hypotheses about the HICE and provide new insights into weathering history during the Hirnantian glaciation.

The Copenhagen Canyon section, situated in Nevada, was deposited in a shallow, platform margin to outer shelf setting^11, 34^. The biostratigraphy of the Copenhagen Canyon section is well established^11, 34^, and this section has been extensively studied and yielded excellent sedimentological and isotopic data^11, 12, 16, 34–37^. The lower part of the Copenhagen Canyon section consists of thick cherty limestone interbedded with thin calcareous mudstone and shale. On the basis of the stratigraphic patterns of lithofacies and fossil records, a sea-level curve has been reconstructed for the Hirnantian Stage at Copenhagen Canyon^11^. Within the *P. pacificus* Biozone, the lithofacies of darker gray, shaley rocks indicate a distinct deepening event, which just preceded the Hirnantian glaciation. Higher up in the section, a subaerial exposure surface is overlain by well sorted, fine quartz arenite, suggesting the maximum sea-level lowstand associated with the second advance of the ice sheet^11, 36^. Immediately overlying the exposure surface, wackestone, packstone, and dark gray lime mudstone with chert suggest post-glacial flooding.

## Results

**Figure 2:**
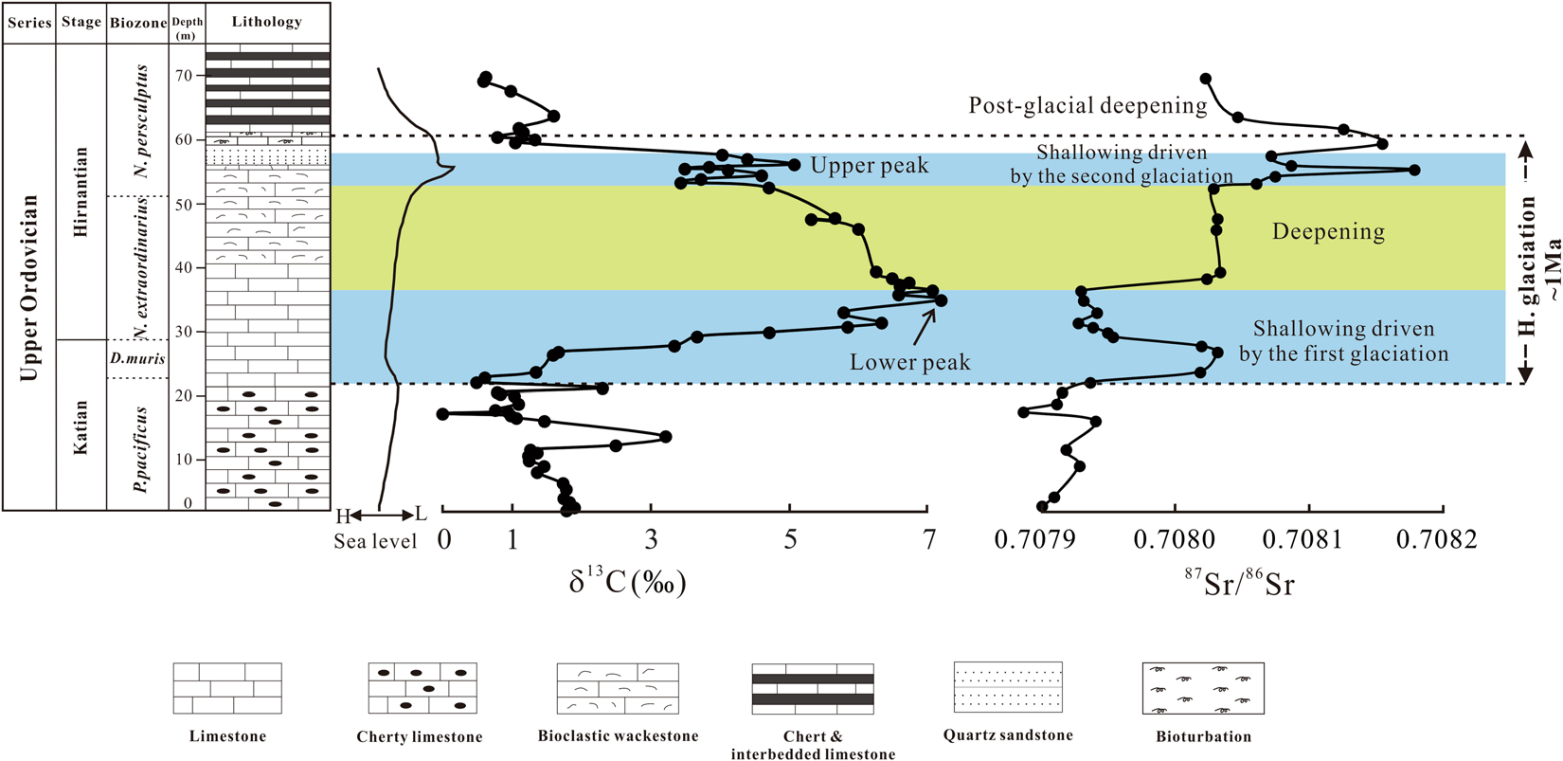
Integrated carbon-strontium isotopic chemostratigraphic profile of the Hirnantian glacial interval at Copenhagen Canyon, Nevada, USA. Two shallowing periods (light blue areas) are separated by an interval of deepening (light green area)^35, 36^. The sea-level curve is from Finney *et al*.^11^.

The δ^13^C values of the upper part of the Katian Stage vary between 0‰ and 3.2‰ and are followed by a typical HICE with a large positive δ^13^C excursion of ~7‰ (Fig. 2, Table S1). Our C-isotopic data are consistent with the previous measurements on the same section^11, 12, 16^. However, we observed a second positive δ^13^C excursion with a peak value of 5.1‰ in the lower *N. persculptus* Biozone, which coincides with the second pulse of the glaciation (Fig. 2, Table S1). Higher stratigraphically, δ^13^C returns to the pre-excursion value of ~0.6‰ in the upper *N. persculptus* Biozone (Fig. 2, Table S1).

The ^87^Sr/^86^Sr profile in the upper Katian is stable with a value of ~0.70791 (Fig. 2, Table S2), which is in good agreement with the ^87^Sr/^86^Sr measured in the correlative strata elsewhere^31–33^. Within the *D. mirus* Biozone, ^87^Sr/^86^Sr values increase sharply from ~0.70791 to 0.70803, which correlates well with the onset of the Hirnantian glaciation as well as the rise of δ^13^C (Fig. 2, Table S2). The ^87^Sr/^86^Sr values decline to 0.70793 in the lower *N. extraordinarius* Biozone where δ^13^C values reach the maximum of 7.2‰ (Fig. 2, Table S2). Stratigraphically higher, the ^87^Sr/^86^Sr ratio increases abruptly to 0.70803 and is then constant in the upper *N. extraordinarius* Biozone, which corresponds to the interglacial deepening as well as the decrease of δ^13^C (Fig. 2, Table S2).

Like the isotopic signature during the first pulse of the glaciation, an increase of ^87^Sr/^86^Sr ratio to 0.70818 followed by a decrease to 0.70807 coincides with the shallowing driven by the second pulse of the glaciation as well as the second positive C-isotopic excursion (Fig. 2, Table S2). The postglacial flooding in the upper *N. persculptus* Biozone coincides with an abrupt ^87^Sr/^86^Sr increase to 0.70815 and subsequent decrease to 0.70802 (Fig. 2, Table S2).

## Discussion

### Assessment of Diagenetic Alteration of Seawater ^87^Sr/^86^Sr

A critical evaluation of diagenetic alteration of seawater ^87^Sr/^86^Sr ratios from bulk carbonates is prerequisite to interpretation of significant changes in ^87^Sr/^86^Sr. Diagenetic alteration can cause depletion in Sr and enrichment in Mn (ref. 19) and thus Sr concentration of bulk carbonate is commonly used as a reliable indicator of diagenetic alteration^17, 20, 30, 38^. By paired ^87^Sr/^86^Sr measurements of Ordovician bulk carbonate and well-preserved conodont apatite, Edwards *et al*.^30^ concluded that the primary seawater ^87^Sr/^86^Sr can be faithfully preserved in bulk carbonate with Sr content >300 ppm.

All the samples we analyzed yield Sr concentrations >300 ppm except one sample with Sr content of 294 ppm, and 15 out of 33 samples contain Sr >1000 ppm. In addition, Mn contents for all samples are <120 ppm (26 out of 33 samples <40 ppm) and all ratios of Sr/Mn >4.9 (23 out of 33 samples >20). The high Sr concentration of >300 ppm, low Mn contents, and the consistent ^87^Sr/^86^Sr ratios of the Katian carbonates with the previous analyses suggest that the primary Hirnantian seawater ^87^Sr/^86^Sr values are well preserved at Copenhagen Canyon.

### Modeling ^87^Sr/^86^Sr and δ^13^C: Implications for perturbations of carbon and strontium cycling

The marine ^87^Sr/^86^Sr ratio is predominantly determined by fluxes from rivers and seafloor hydrothermal exchanges at mid-oceanic ridges^17, 19, 39^. The ^87^Sr/^86^Sr ratios of river waters are highly variable (~0.711 or higher) and dependent on the relative contributions of continental weathering sources^40, 41^. For example, carbonates contain high Sr concentrations (up to 1000 ppm) but low ^87^Sr/^86^Sr ratios ranging from 0.706 to 0.709 (ref. 42). In contrast, old crustal terrains with greater resistance to weathering have lower Sr concentrations but high ^87^Sr/^86^Sr ratios of >0.710 (refs 39, 42). Moreover, basaltic volcanic rocks are characterized by nonradiogenic ^87^Sr/^86^Sr values of ~0.704 (refs 33, 40) and hydrothermal flux has a nearly homogeneous ^87^Sr/^86^Sr value of ~0.703 (ref. 43).

We use a numerical box model to simulate the δ^13^C and ^87^Sr/^86^Sr variations against the isotopic records in the Copenhagen Canyon section (see details in Table S3). Briefly, the model is composed of a system of three fundamental equations that illustrate the mass balances and isotope mass balances for Sr and C in the ocean. The mass balance equation can be expressed as equation (1),

1 $$\frac{d{M}_{i}^{SW}}{dt}={F}_{in,i}-{F}_{out,i}$$

where ${M}_{i}^{SW}$ represents the mass of element *i* in the ocean, *t* is time, ${F}_{in,i}$ and ${F}_{out,i}$ are total input and output fluxes of element *i* respectively.

For the Sr isotopic ratio ${R}_{Sr}^{j}={({}^{87}{\rm{S}}{\rm{r}}/{}^{86}{\rm{S}}{\rm{r}})}^{j}$, the rate of change of the seawater ^87^Sr/^86^Sr is given by equation (2),

2 $$\frac{d{R}_{Sr}^{SW}}{dt}=\frac{{F}_{in,Sr}({R}_{Sr}^{in}-{R}_{Sr}^{SW})}{{M}_{Sr}^{SW}}$$

where ${R}_{Sr}^{SW}$ and ${R}_{Sr}^{in}$ are ^87^Sr/^86^Sr ratios of seawater and input fluxes respectively.

For the carbon isotope systematics, the rate of change of the seawater δ^13^C is shown in equation (3),

3 $$\frac{d\delta {}^{13}{C}^{SW}}{dt}=\frac{{F}_{in,C}(\delta {}^{13}{C}^{in}-\delta {}^{13}C{}^{SW})-{J}_{out,ORG}^{C}{{\rm{\Delta }}}_{ORG-SW}}{{M}_{C}^{SW}}$$

where δ^13^C^*SW*^ and δ^13^C^*in*^ are the carbon isotopic compositions of seawater and input fluxes, ${J}_{out,ORG}^{C}$ is output flux as organic carbon buried in sediments, Δ_*ORG-SW*_ is the carbon isotopic fractionation between output flux of organic carbon and seawater reservoir.

In our simulation, we focus on changes in fluxes and isotopic compositions of the continental inputs derived from weathering of carbonates and silicates. The large positive δ^13^C excursion is assumed to start at *t* = 0 and changes in fluxes and isotopic compositions are all applied instantaneously to obtain the first-order estimate of the impacts on marine ^87^Sr/^86^Sr and δ^13^C induced by the glacial-interglacial cycles.

**Figure 3:**
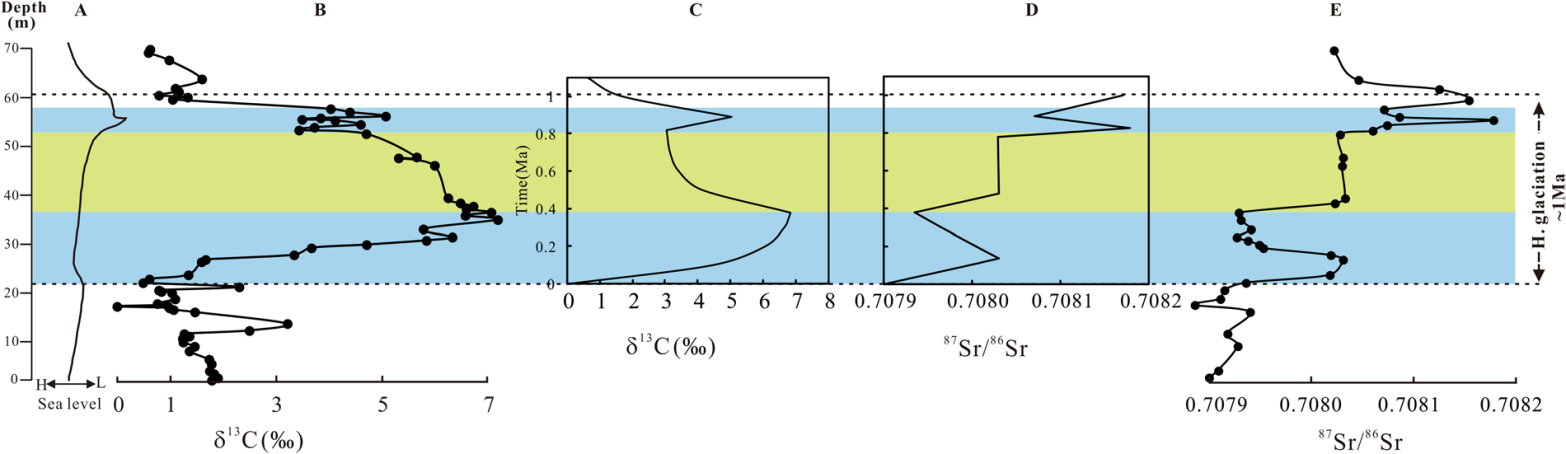
Sea-level curve (**A**), δ^13^C and ^87^Sr/^86^Sr data from Copenhagen Canyon section (**B**,**E**), and the numerical model simulations of marine δ^13^C and ^87^Sr/^86^Sr as responses to increased weathering of carbonate during the Hirnantian sea-level drawdown (**C**,**D**). The reconstructed sea-level curve is from Finney *et al*.^11^.

The original δ^13^C of continental inputs (δ^13^C_*in*, *CONT*_) is ~−7‰, which includes 28% contributions from weathering of organic matter (δ^13^C_*in*, *ORG*_ = −25‰) and 72% from weathered carbonates (δ^13^C_*in*, *CARB*_ = 0‰)^16^. However, the δ^13^C_*in, CONT*_ would increase to ~0‰ resulting from increased exposure and weathering of carbonate platforms during the glacio-eustatically controlled sea-level drawdown. The sea-level fall also led to the restricted seawater circulation between the Martin Ridge basin and the open ocean^12, 36^, and thus the contribution of C-flux from the open ocean would have been dramatically diminished by inputs of continental C-fluxes. Consequently, we obtained a positive excursion in δ^13^C of ~7‰ at ~0.38 Ma with timing and magnitude fitting well with the δ^13^C excursion in the Copenhagen Canyon section (Fig. 3B and C).

The ensuing decline of HICE was driven by a reduction in δ^13^C_*in, CONT*_ (from 0‰ to −4‰, Table S3), reflecting ice sheet retreat, sea-level rise, transgression, and the accompanied reduction of carbonate weathering. The second, higher, positive δ^13^C excursion (from 3.4‰ to 5.1‰, Fig. 3B and C) also resulted from an increase in δ^13^C_*in, CONT*_ (from −4‰ to 0‰, Table S3) that was induced by the re-exposure of carbonate platforms associated with the second glacial advance. Higher in the section, δ^13^C returned to the pre-excursion value coincident with the post-glacial flooding.

Our modeling of δ^13^C excursions is apparently consistent with the δ^13^C records from the Copenhagen Canyon section (Fig. 3). Therefore, our data and modeling suggest that, without a change of organic carbon burial rate, an increase in δ^13^C of the continental input C-flux can result in large positive δ^13^C excursions in the epeiric sea.

We can further test the weathering hypothesis by simulating ^87^Sr/^86^Sr variations because seawater ^87^Sr/^86^Sr ratio is an effective proxy of continental weathering. Figure 3D illustrates the simulation of the Hirnantian seawater ^87^Sr/^86^Sr oscillations by changing Sr-sources and their isotopic compositions. In our simulation, a good fit with the sharp increase in ^87^Sr/^86^Sr from ~0.70791 to 0.70803 near the base of the Hirnantian Stage is achieved when the ^87^Sr/^86^Sr ratios of silicates weathering flux increase from 0.721 to 0.7315 (Fig. 3, Table S3). An increased flux of silicate weathering (${F}_{in,SIL}^{Sr}$) can also lead to a rise of seawater ^87^Sr/^86^Sr ratios because both flux and ^87^Sr/^86^Sr ratio (${R}_{in,SIL}^{Sr}$) can affect the product ${F}_{in,SIL}^{Sr}\times {R}_{in,SIL}^{Sr}$ that represents chemical weathering rate of silicates. The increase in ^87^Sr/^86^Sr ratios of silicate weathering flux in our simulation is consistent with geological processes at the beginning of the Hirnantian glaciation. The enhanced mechanical erosion driven by glacial grinding and abrasion may have produced fine-grained Rb-rich glacial till with minerals such as biotite^24, 25^, which possesses radiogenic ^87^Sr/^86^Sr ratios of ≥0.9245 (ref. 44). Also, global cooling can independently promote the preferential weathering of biotite^26^. The sudden influx of these detrital phases to epeiric seas would cause an abrupt increase of seawater ^87^Sr/^86^Sr ratios. The abrupt increase of seawater ^87^Sr/^86^Sr along with gradual increase of δ^13^C near the basal Hirnantian Stage suggest that silicate minerals were the dominate source of Sr at the beginning of the glaciation despite gradual exposure of carbonate platforms.

To simulate the following sharp drop in ^87^Sr/^86^Sr, we increased the proportional contribution of carbonate weathering to the total Sr input fluxes from 36.4% to 61.7%, similar to our simulation of the δ^13^C rise during the same interval (Table S3). Evidently, the increasing Sr contents in this interval (Fig. S2), resulting from weathering of more exposed shelf carbonates due to glacial expansion, support the modeling analyses. The modeling also indicates that the decline to a minimum of ^87^Sr/^86^Sr ratios would take ~0.38 Ma (Fig. 3D).

The following increase to 0.70803 and the steady ^87^Sr/^86^Sr trend in the middle Hirnantian corresponded with the interglacial period of ice sheet recession that would have resulted in a sea-level rise and submergence of the carbonate platforms and also would have left behind extensive, fresh, fine-grained moraines highly susceptible to weathering, especially with warmer temperature and much more melt water^24, 28^. The initial weathering of these fragile materials would elevate ^87^Sr/^86^Sr ratios of silicate weathering flux from 0.721 to 0.7315, the same as the onset of the glaciation (Table S3) owing to the preferential weathering of biotite, which is a common Rb-rich mineral with high ^87^Sr/^86^Sr ratios^24, 25, 44^. However, the ^87^Sr/^86^Sr ratios would decrease with the increasing age of the materials being weathered^24, 28^. As a result, there would be a weathering “spike” immediately following the deglaciation and a steady ^87^Sr/^86^Sr trend at 0.70803 in the middle Hirnantian (Fig. 3D and E).

Thereafter, the advance of the second glaciation led to the same changes of C and Sr cycling as that of the first glaciation. The rise of seawater ^87^Sr/^86^Sr at the beginning and end of the second glaciation would also be driven by increase in ^87^Sr/^86^Sr of silicate weathering flux (Table S3) associated with preferential weathering of biotite. Whereas, the glacial maximum would be consistent with positive δ^13^C excursion and coeval with drop of ^87^Sr/^86^Sr that was caused by enhanced carbonate weathering (Table S3).

However, the modeling results show that the increase of ^87^Sr/^86^Sr from 0.70803 to 0.70818 associated with the onset of the second glaciation would have taken ~0.17 Ma owing to the long residence time of Sr (~2.7 Ma) in seawater^18^. This is inconsistent with our observation that the ^87^Sr/^86^Sr dropped to 0.70807 at the same time as the peak in the second positive δ^13^C excursion at ~0.89 Ma (Fig. 3B and E). The only way to achieve synchronous shifts in δ^13^C and ^87^Sr/^86^Sr at ~0.89 Ma is to decrease the residence time of Sr by decreasing the mass of Sr in the reservoir, by increasing the input and output Sr-fluxes, or by a combination of the both. It is reasonable to significantly decrease the residence time of Sr in an epeiric sea due to smaller water masses and higher flux of weathered Sr from carbonates during the Hirnantian eustatic lowstand. Using a residence time of 0.83 Ma, a good fit of δ^13^C and ^87^Sr/^86^Sr shifts during the second glaciation is obtained (Fig. 3).

The second deglaciation led to a weathering “spike” of ^87^Sr/^86^Sr values increasing to 0.70815 rapidly and then declining to 0.70802 (Fig. 3E). These ^87^Sr/^86^Sr patterns are the same as those of the first glaciation advance, and are essentially controlled by the changes in the weathering rates of carbonates and biotite associated with the waxing and waning of the Hirnantian glaciations. Thus the Hirnantian glaciation within ~1 Ma correlates qualitatively with changes in δ^13^C and seawater ^87^Sr/^86^Sr.

The glacial advances and associated changes in weathering regime, the mass fraction and isotopic ratios of different input fluxes chosen, together with local effects of C and Sr-cycling in the epeiric sea, offer an internal consistency to the observed C and Sr isotopes records in the Copenhagen Canyon section. The C and Sr isotopic data support the hypothesis that the Hirnantian positive δ^13^C excursion resulted from enhanced carbonate weathering during glacioeustatic sea-level drawdown.

## Methods

For δ^13^C analysis, approximately 150 μg samples were reacted with ~103% phosphoric acid at 70 °C in a Kiel IV carbonate device connected to a Thermo Scientific MAT 253 mass spectrometer. The carbon isotopic compositions are reported in the standard delta (δ) notation as permil (‰) deviations from Vienna PeeDee Belemnite (V-PDB), with external precision of ~0.05‰ (1σ) based on duplicate analyses of an internal standard. For analysis of ^87^Sr/^86^Sr ratio, carbonate powders of ~120 mg were dissolved in 30% acetic acid at room temperature to avoid dissolution of the non-carbonates. The solution then was centrifuged, evaporated and re-dissolved in 2.5 N HCl, standard cation-exchange procedures were performed to purify Sr from matrix ions and ^87^Sr/^86^Sr ratios were analyzed using a Thermo Scientific Triton thermal ionization mass spectrometer, following methods outlined in Lin *et al*.^45^. The reported ^87^Sr/^86^Sr ratios were corrected for instrumental mass fractionation using a ratio of ^86^Sr/^88^Sr = 0.1194. Trace metal concentrations (Mn, Sr) were measured using an ICP AES instrument with a reproducibility of ±10% (2σ).

## Electronic supplementary material

Supplementary Information
