# Supplementary material for: 87Sr/86Sr evidence from the epeiric Martin Ridge Basin for enhanced carbonate weathering during the Hirnantian
Source: Sci Rep. 2017 Sep 12;7:11348. doi: 10.1038/s41598-017-11619-w (PMC5595791; doi:10.1038/s41598-017-11619-w)
Supplement: Supplementary file 1 — Supplementary Information [file 41598_2017_11619_MOESM1_ESM.pdf]

Supplementary Information

$^{87}Sr/^{86}Sr$ evidence from the epeiric Martin Ridge Basin for enhanced carbonate weathering during the Hirnantian

Dongping Hu[1], Xiaolin Zhang[1*], Lian Zhou[2], Stanley C. Finney[3], Yongsheng Liu[2], Danielle Shen[4], Megan Shen[4], Wei Huang[1], Yanan Shen[1]

[1]School of Earth and Space Sciences, University of Science and Technology of China, Hefei 230026, China.

[2]State Key Laboratory of Geological Processes and Mineral Resources, Faculty of Earth Sciences, China University of Geosciences, Wuhan 430074, China.

[3]Department of Geological Sciences, California State University at Long Beach, Long Beach, CA 90840, USA.

[4]University of Maryland, College Park, MD 20742, USA.

*To whom correspondence should be addressed. E-mail: zhxl2012@ustc.edu.cn

Table S1: C and O isotopic data of the Copenhagen Canyon section

Sample NO.	Stage	Lithology	$\delta^{13}C$ (V-PDB,‰)	$\delta^{18}O$ (V-PDB,‰)	Depth(m)
COK-71.2	Hirnantian	limestone intercalated with chert	0.62	-7.26	71.2
COK-70.5	Hirnantian	limestone intercalated with chert	0.58	-7.53	70.5
COK-68.9	Hirnantian	limestone intercalated with chert	0.98	-7.21	68.9
COK-64.8	Hirnantian	limestone intercalated with chert	1.59	-6.68	64.8
COK-62.8	Hirnantian	limestone intercalated with chert	1.09	-8.25	62.8
COK-62.1	Hirnantian	limestone intercalated with chert	1.16	-8.25	62.1
COK-61.3	Hirnantian	limestone intercalated with chert	0.78	-8.57	61.3
COK-60.9	Hirnantian	limestone intercalated with chert	1.32	-8.70	60.9
COK-60.4	Hirnantian	limestone intercalated with chert	1.04	-9.79	60.4
COK-58.4	Hirnantian	limestone	4.02	-8.72	58.4
COK-57.7	Hirnantian	limestone	4.38	-8.81	57.7
COK-56.8	Hirnantian	limestone	5.06	-7.71	56.8
COK-56.4	Hirnantian	limestone	3.83	-7.97	56.4
COK-56.1	Hirnantian	limestone	3.48	-8.28	56.1
COK-55.9	Hirnantian	limestone	4.11	-7.71	55.9
COK-55	Hirnantian	limestone	4.59	-7.06	55.0
COK-54.4	Hirnantian	limestone	3.71	-6.06	54.4
COK-53.8	Hirnantian	limestone	3.42	-8.29	53.8
COK-53.0	Hirnantian	limestone	4.69	-7.74	53.0
COK-48.0	Hirnantian	limestone	5.64	-7.28	48.0
COK-47.8	Hirnantian	limestone	5.30	-6.17	47.8
COK-46.2	Hirnantian	limestone	5.98	-7.05	46.2
COK-39.2	Hirnantian	limestone	6.24	-12.86	39.2
COK-38.1	Hirnantian	limestone	6.47	-7.81	38.1
COK-37.4	Hirnantian	limestone	6.71	-7.88	37.4
COK-36.8	Hirnantian	limestone	6.58	-7.42	36.8
COK-36.1	Hirnantian	limestone	7.05	-6.40	36.1
COK-35.4	Hirnantian	limestone	6.56	-7.08	35.4
COK-34.5	Hirnantian	limestone	7.17	-7.04	34.5
COK-32.5	Hirnantian	limestone	5.77	-7.97	32.5
COK-30.8	Hirnantian	limestone	6.32	-5.85	30.8
COK-30.1	Hirnantian	limestone	5.83	-4.84	30.1
COK-29.2	Hirnantian	limestone	4.70	-6.03	29.2
COK-28.5	Hirnantian	limestone	3.66	-6.33	28.5
COK-27	Katian	limestone	3.33	-6.56	27.0
COK-26	Katian	limestone	1.66	-4.36	26.0

Continued

COK-25.5	Katian	limestone	1.58	-2.88	25.5
COK-22.7	Katian	limestone	1.34	-6.39	22.7
COK-21.8	Katian	limestone	0.60	-6.05	21.8
COK-21.0	Katian	cherty limestone	0.48	-5.84	21.0
COK-20.1	Katian	cherty limestone	2.30	-15.01	20.1
COK-19.4	Katian	cherty limestone	0.78	-6.27	19.4
COK-19.1	Katian	cherty limestone	0.83	-4.19	19.1
COK-18.8	Katian	cherty limestone	1.03	-5.53	18.8
COK-17.5	Katian	cherty limestone	1.09	-5.20	17.5
COK-16.5	Katian	cherty limestone	0.75	-4.60	16.5
COK-16.2	Katian	cherty limestone	0.94	-5.23	16.2
COK-15.9	Katian	cherty limestone	-0.01	-5.15	15.9
COK-15.6	Katian	cherty limestone	0.98	-4.10	15.6
COK-15.2	Katian	cherty limestone	1.06	-5.13	15.2
COK-14.7	Katian	cherty limestone	1.46	-4.74	14.7
COK-12.2	Katian	cherty limestone	3.21	-14.69	12.2
COK-10.7	Katian	cherty limestone	2.49	-6.49	10.7
COK-10.0	Katian	cherty limestone	1.26	-5.05	10.0
COK-9.5	Katian	cherty limestone	1.36	-4.93	9.5
COK-9.0	Katian	cherty limestone	1.23	-5.25	9.0
COK-8.2	Katian	cherty limestone	1.24	-5.60	8.2
COK-7.3	Katian	cherty limestone	1.45	-5.89	7.3
COK-6.30	Katian	cherty limestone	1.36	-6.13	6.3
COK-4.5	Katian	cherty limestone	1.73	-4.55	4.5
COK-3.5	Katian	cherty limestone	1.77	-6.19	3.5
COK-2.00	Katian	cherty limestone	1.74	-5.80	2.0
COK-1.4	Katian	cherty limestone	1.82	-4.99	1.4
COK-1.05	Katian	cherty limestone	1.81	-5.17	1.1
COK-0.5	Katian	cherty limestone	1.89	-4.62	0.5
COK-0	Katian	cherty limestone	1.78	-5.53	0.0

Table S2: Sr isotopic data and Sr and Mn concentrations of the Copenhagen Canyon section

Sample NO.	Stage	$^{87}Sr/^{86}Sr$	±2σ	Sr con(ppm)	Mn con(ppm)	Sr/Mn	Depth(m)
COK-71.2	Hirnantian	0.708022	0.000005	614.8	18.4	33.4	71.2
COK-64.8	Hirnantian	0.708046	0.000008	532.1	22.7	23.4	64.8
COK-62.8	Hirnantian	0.708125	0.000005	313.5	59.4	5.3	62.8
COK-60.4	Hirnantian	0.708154	0.000007	293.9	34.2	8.6	60.4
COK-58.4	Hirnantian	0.708071	0.000005	407.5	30.2	13.5	58.4
COK-56.8	Hirnantian	0.708086	0.000005	342.4	21.7	15.8	56.8
COK-56.1	Hirnantian	0.708178	0.000006	300.7	29.0	10.4	56.1
COK-55	Hirnantian	0.708074	0.000007	436.7	29.4	14.9	55.0
COK-53.8	Hirnantian	0.70806	0.000005	361.5	23.2	15.6	53.8
COK-53.0	Hirnantian	0.708028	0.000005	468.4	20.2	23.2	53.0
COK-48	Hirnantian	0.708031	0.000006	403.3	16.4	24.5	48.0
COK-46.2	Hirnantian	0.708030	0.000005	390.1	18.8	20.8	46.2
COK-39.2	Hirnantian	0.708033	0.000005	506.2	19.1	26.5	39.2
COK-38.1	Hirnantian	0.708023	0.000004	485.1	16.6	29.2	38.1
COK-36.1	Hirnantian	0.707929	0.000003	1105.7	15.6	70.8	36.1
COK-34.5	Hirnantian	0.707931	0.000004	1378.9	17.1	80.4	34.5
COK-32.5	Hirnantian	0.707941	0.000004	1599.7	47.2	33.9	32.5
COK-30.8	Hirnantian	0.707927	0.000004	1832.0	31.8	57.5	30.8
COK-30.1	Hirnantian	0.707938	0.000007	1243.1	31.7	39.2	30.1
COK-29.2	Hirnantian	0.707949	0.000006	1062.9	50.2	21.2	29.2
COK-28.5	Hirnantian	0.707953	0.000004	1074.4	47.3	22.7	28.5
COK-27	Katian	0.708019	0.000005	1040.3	34.8	29.9	27.0
COK-26	Katian	0.708031	0.000003	570.4	116.8	4.9	26.0
COK-22.7	Katian	0.708018	0.000003	711.5	59.3	12.0	22.7
COK-21.0	Katian	0.707936	0.000005	735.6	41.2	17.8	21.0
COK-19.4	Katian	0.707915	0.000005	1375.2	28.9	47.6	19.4
COK-17.5	Katian	0.707911	0.000004	1292.9	21.6	59.8	17.5
COK-16.2	Katian	0.707886	0.000004	1387.0	25.2	55.0	16.2
COK-14.7	Katian	0.707940	0.000006	926.7	37.6	24.7	14.7
COK-10.0	Katian	0.707918	0.000004	1198.3	19.5	61.6	10.0
COK-7.3	Katian	0.707928	0.000007	1010.3	19.9	50.8	7.3
COK-2.00	Katian	0.707909	0.000005	1104.1	24.7	44.7	2.0
COK-0.5	Katian	0.707900	0.000003	1216.3	21.4	56.9	0.5

Table S3: Equations, parameters and descriptions of our modeling calculation

**Equations**
$\frac{dM_i^{SW}}{dt} = F_{in,i} - F_{out,i}$
$\frac{dR_{Sr}^{SW}}{dt} = \frac{F_{in,Sr}(R_{Sr}^{in} - R_{Sr}^{SW})}{M_{Sr}^{SW}}$
$\frac{d\delta^{13}C^{SW}}{dt} = \frac{F_{in,C}(\delta^{13}C^{in} - \delta^{13}C^{SW}) - J_{out,ORG}^{C}\Delta_{ORG-SW}}{M_C^{SW}}$
$R_{Sr}^{SW}(t) = (f_{in,DM}^{Sr} \times R_{in,DM}^{Sr} + f_{in,SIL}^{Sr} \times R_{in,SIL}^{Sr} + f_{in,CARB}^{Sr} \times R_{in,CARB}^{Sr})$ $+[R_{Sr}^{SW}(t_0) - (f_{in,DM}^{Sr} \times R_{in,DM}^{Sr} + f_{in,SIL}^{Sr} \times R_{in,SIL}^{Sr} + f_{in,CARB}^{Sr} \times R_{in,CARB}^{Sr})] \times e^{\frac{t_0 - t}{\tau_{Sr}^{SW}}}$
$\delta^{13}C^{SW}(t) = (\delta^{13}C^{in} - f_{out,ORG}^{C}\Delta_{ORG-SW})$ $+[\delta^{13}C^{SW}(t_0) + f_{out,ORG}^{C}\Delta_{ORG-SW} - \delta^{13}C^{in}] \times e^{\frac{t_0 - t}{\tau_C^{SW}}}$

**Parameters**	**Descriptions**	**Initial values**	**References and notes**
M_i^{SW}	the mass of element *i* in the ocean		
$F_{in,i}$	total input flux of element *i*		
$F_{out,i}$	total output flux of element *i*		
R_{Sr}^{SW}	$^{87}Sr/^{86}Sr$ ratios of seawater	0.7079	Saltzman *et al.*[31]; Shields *et al.*[32]
$\delta^{13}C^{SW}$	$\delta^{13}C$ value of seawater		
$\delta^{13}C^{in}$	$\delta^{13}C$ value of the total input flux		
$J_{out,ORG}^{C}$	output flux as organic carbon buried in sediments		
Δ_{ORG-SW}	carbon isotopic fractionation between output flux as organic carbon and seawater reservoir	-25‰	Jacobsen and Kaufman[46]; Hayes *et al.*[47]

$f_{in,DM}^{Sr}$	mass fraction of Sr-input flux from depleted mantle (i.e. hydrothermal fluids) and basaltic volcanic rocks	53.3%	Based on the mass balance calculation
$f_{in,SIL}^{Sr}$	mass fraction of Sr-input flux from silicates weathering	20.3%	Based on the mass balance calculation
$f_{in,CARB}^{Sr}$	mass fraction of Sr-input flux from carbonates weathering	26.4%	Based on the mass balance calculation
$R_{in,DM}^{Sr}$	$^{87}Sr/^{86}Sr$ ratio of depleted mantle and basaltic volcanic rocks flux	0.703	Allègre *et al.*[40]; Albarède *et al.*[43]
$R_{in,SIL}^{Sr}$	$^{87}Sr/^{86}Sr$ ratio of flux from silicates weathering	0.721	Allègre *et al.*[40]
$R_{in,CARB}^{Sr}$	$^{87}Sr/^{86}Sr$ ratio of flux from carbonates weathering	0.7079	Saltzman *et al.*[31]; this study
τ_{Sr}^{SW}	residence time of Sr in seawater	2.7Ma	Capo and Depaolo[18]
τ_{C}^{SW}	residence time of C in seawater	0.1Ma	Kump[48]

Model results of changes in C isotopic compositions relative to pre-excursion value plotted in Fig. 3C.

Time(Ma)	$\delta^{13}C_{in,CONT}$ (‰)	$\Delta^{13}C^{in}$ (‰)	$\Delta^{13}C^{SW}$ (‰)
0	0	7	0
0.1	0	7	4.424844
0.2	0	7	6.052653
0.3	0	7	6.651491
0.35	0	7	6.788618
0.38	0	7	6.843405
0.5	-4	3	4.157611
0.6	-4	3	3.425861
0.7	-4	3	3.156666
0.82	-4	3	3.047187
0.89	0	7	5.037091
1	-7	0	1.676702
1.1	-7	0	0.616824

Model results of Sr isotopic compositions plotted in Fig. 3D based on parameters in Table S3

Time (Ma)	$f_{in,DM}^{Sr}$	$R_{in,DM}^{Sr}$	$f_{in,SIL}^{Sr}$	$R_{in,SIL}^{Sr}$	$f_{in,CARB}^{Sr}$	$R_{in,CARB}^{Sr}$	τ_{Sr}^{SW} (Ma)	R_{Sr}^{SW}
0	0.433	0.703	0.203	0.7315	0.364	0.7079	2.7	0.707900
0.1	0.433	0.703	0.203	0.7315	0.364	0.7079	2.7	0.7079971
0.136	0.433	0.703	0.203	0.7315	0.364	0.7079	2.7	0.7080311
0.2	0.333	0.703	0.05	0.721	0.617	0.7079	2.7	0.7080052
0.3	0.333	0.703	0.05	0.721	0.617	0.7079	2.7	0.7079658
0.38	0.333	0.703	0.05	0.721	0.617	0.7079	2.7	0.7079354
0.4	0.433	0.703	0.203	0.7315	0.364	0.7079	2.7	0.7079548
0.48	0.433	0.703	0.203	0.7315	0.364	0.7079	2.7	0.7080312
0.6	0.433	0.703	0.171	0.721	0.396	0.7079	2.7	0.7080306
0.7	0.433	0.703	0.171	0.721	0.396	0.7079	2.7	0.7080302
0.78	0.433	0.703	0.171	0.721	0.396	0.7079	2.7	0.7080298
0.8	0.433	0.703	0.203	0.7315	0.364	0.7079	0.83	0.7080903
0.83	0.433	0.703	0.203	0.7315	0.364	0.7079	0.83	0.7081783
0.86	0.31	0.703	0.02	0.721	0.67	0.7079	0.83	0.7081238
0.89	0.31	0.703	0.02	0.721	0.67	0.7079	0.83	0.7080712
0.95	0.433	0.703	0.203	0.7315	0.364	0.7079	2.7	0.7081261
1	0.433	0.703	0.203	0.7315	0.364	0.7079	2.7	0.7081709

Note: The change of $f_{in,DM}^{Sr}$ during the calculation is response of restricted water circulation and coverage of ice sheets during the glaciation interval. The contribution of Sr flux from depleted mantle is ~10% (ref. 40), that is discounted during the glaciation owing to restricted water exchange. Consequently, the mantle-derived Sr flux is predominantly controlled by weathering of basaltic volcanic rocks and would decrease as a result of ice coverage during the glacial maximum.

Figure legends

Fig. S1: Marine $^{87}Sr/^{86}Sr$ curve through Mid-Upper Ordovician modified from Young *et al.*[33]. The best estimation of marine $^{87}Sr/^{86}Sr$ trend is illustrated by black dashed line[33]. The additional fine structure of Hirnantian seawater $^{87}Sr/^{86}Sr$ record is from this study. H- Hirnantian.

Fig. S2: Relationship between $^{87}Sr/^{86}Sr$ and Sr concentrations of the samples. The lower $^{87}Sr/^{86}Sr$ corresponding with higher Sr concentrations indicates that the low $^{87}Sr/^{86}Sr$ ratios of seawater were driven by enhanced weathering of carbonate with relatively unradiogenic Sr isotopic compositions (~0.7079) and higher Sr concentrations.

Fig. S3: Scatter diagram of Mn versus Sr content in the bulk carbonates. The absence of systematic relationship between Mn and Sr, high Sr and low Mn concentrations suggest little or no diagenetic alteration.

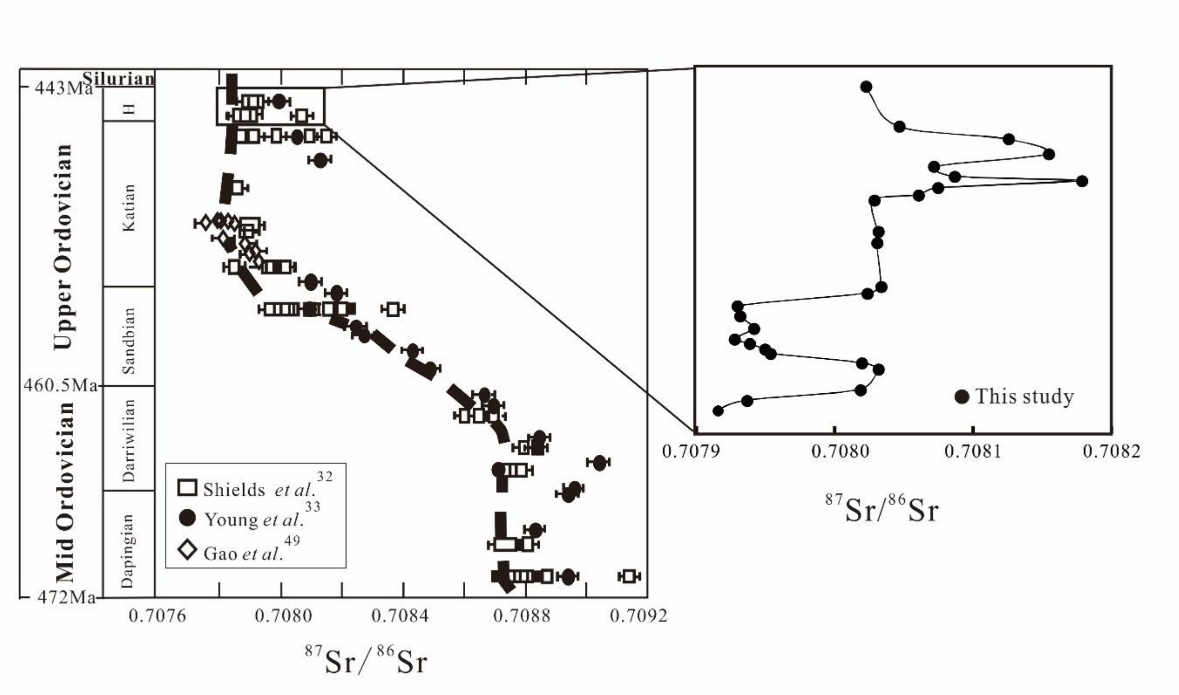
Silurian
443Ma
460.5Ma
472Ma
H
Katian
Sandbian
Darriwilian
Dapingian
Upper Ordovician
Mid Ordovician
Shields et al.[32]
Young et al.[33]
Gao et al.[49]
0.7076
0.7080
0.7084
0.7088
0.7092
87Sr/86Sr
This study
0.7079
0.7080
0.7081
0.7082
87Sr/86Sr

Fig. S1

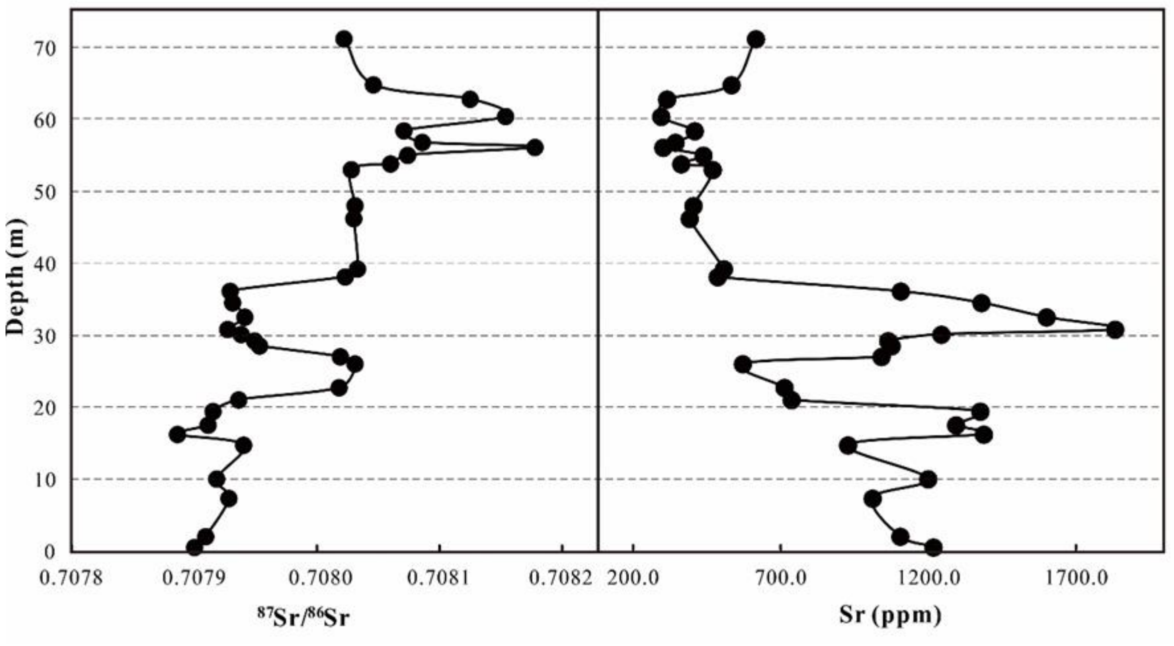
Depth (m)
70
60
50
40
30
20
10
0
0.7078
0.7079
0.7080
0.7081
0.7082
87Sr/86Sr
200.0
700.0
1200.0
1700.0
Sr (ppm)

Fig. S2

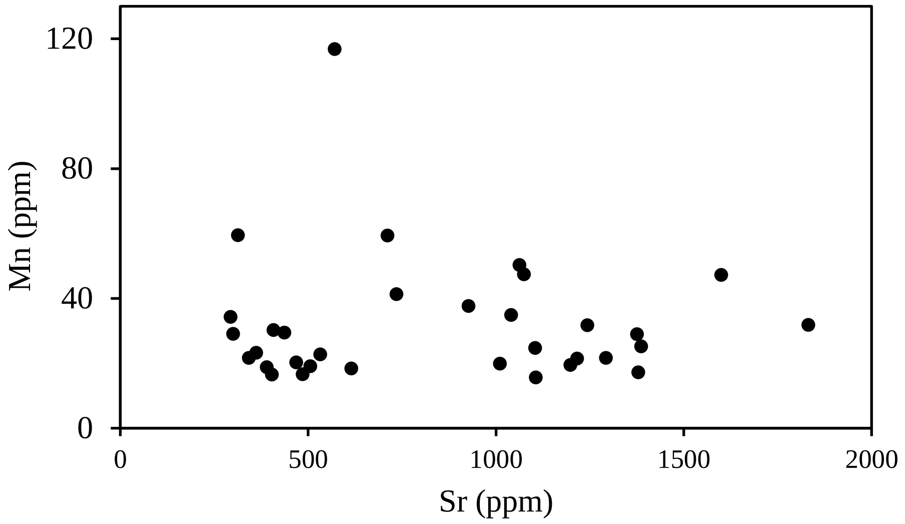

Fig. S3

Additional References

46. Jacobsen, S. B. & Kaufman, A. J. The Sr, C and O isotopic evolution of Neoproterozoic seawater. *Chem. Geol.* **161**, 37-57 (1999).

47. Hayes, J. M., Strauss, H. & Kaufman, A. J. The abundance of ^{13}C in marine organic matter and isotopic fractionation in the global biogeochemical cycle of carbon during the past 800 Ma. *Chem. Geol.* **161**, 103-125 (1999).

48. Kump, L. R. Interpreting carbon-isotope excursions: Strangelove oceans. *Geology* **19**, 299-302 (1991).

49. Gao, G., Dworkin, S. I., Land, L. S. & Elmore, R. D. Geochemistry of Late Ordovician Viola Limestone, Oklahoma: Implications for Marine Carbonate Mineralogy and Isotopic Compositions. *The Journal of Geology* **104**, 359-367 (1996).
